# Towards an Evidence-Based Recommendation for a Balanced Breakfast—A Proposal from the International Breakfast Research Initiative

**DOI:** 10.3390/nu10101540

**Published:** 2018-10-18

**Authors:** Michael J. Gibney, Susan I. Barr, France Bellisle, Adam Drewnowski, Sisse Fagt, Sinead Hopkins, Barbara Livingstone, Gregorio Varela-Moreiras, Luis Moreno, Jessica Smith, Florent Vieux, Frank Thielecke, Gabriel Masset

**Affiliations:** 1 Institute of Food and Health, University College Dublin, Dublin D04 V1W8, Ireland; mike.gibney@ucd.ie; 2 Department of Food, Nutrition & Health, University of British Columbia, Vancouver, BC V6T 1Z4, Canada; susan.barr@ubc.ca; 3 Nutri Psy Consult, 91 rue de la Santé, 75013 Paris, France; bellisle@uren.smbh.univ-paris13.fr; 4 Center for Public Health Nutrition, University of Washington, Seattle, WA 98195-3410, USA; adrewnow@fredhutch.org; 5 Division for Risk Assessment and Nutrition, The National Food Institute, Technical University of Denmark, 2800 Kgs. Lyngby, Denmark; sisfa@food.dtu.dk; 6 Cereal Partners Worldwide, CH-1350 Orbe, Switzerland; sinead.hopkins@rd.nestle.com; 7 Nutrition Innovation Centre for Food and Health (NICHE), Ulster University, Coleraine BT52 1SA, UK; mbe.livingstone@ulster.ac.uk; 8 Department of Pharmaceutical and Health Sciences, Faculty of Pharmacy, CEU San Pablo University, 28668 Madrid, Spain; gvarela@ceu.es; 9 GENUD (Growth, Exercise, Nutrition and Development) Research Group, Universidad de Zaragoza, Instituto de Investigación Sanitaria Aragón (IIS Aragón), Instituto Agroalimentario de Aragón (IA2), Centro de Investigación Biomédica en red Fisiopatología de la Obesidad y Nutrición (CIBEROBN), 50009 Zaragoza, Spain; lmoreno@unizar.es; 10 Bell Institute of Health and Nutrition, General Mills, Minneapolis, MN 55427-3870, USA; Jessica.Smith@genmills.com; 11 MS-Nutrition, 13385 Marseille CEDEX 5, France; florent.vieux@ms-nutrition.com; 12 Swiss Distance University of Applied Sciences, Althardstrasse 60, Regendorf-Zürich CH-8105, Switzerland; frank.b.thielecke@gmail.com

**Keywords:** breakfast, IBRI, nutrient recommendations

## Abstract

The International Breakfast Research Initiative (IBRI) set out to derive nutritional recommendations for a balanced breakfast using a standardized analysis of national nutrition surveys from Canada, Denmark, France, Spain, UK and the US. In all countries, the frequency of breakfast consumption by age was high and U-shaped with children and older adults having a higher frequency of breakfast consumption. Breakfast contributed 16% to 21% of daily energy intake. In all countries, breakfast was a carbohydrate- and nutrient-rich meal, providing more carbohydrates (including sugars), thiamin, riboflavin, folate, calcium, potassium, and magnesium, and less vitamin A, fats and sodium relative to its contribution to daily energy intakes. Breakfast consumers were stratified by tertiles of the Nutrient Rich Foods (NRF) index, used as a measure of diet quality. Breakfast intakes associated with the top tertile of NRF, along with the Codex Alimentarius international food standards and World Health Organization (WHO) diet guidelines, were used to derive the proposed nutrient recommendations. The goal was to preserve the nutrient density of existing breakfasts, while addressing concerns regarding added sugars, saturated fats, dietary fiber, and vitamin D. This initiative is unique in seeking to derive nutrient recommendations for a specific meal using the observed nutritional profile of such meal.

## 1. Introduction

Breakfast is widely considered to be a key component of a healthy diet. Regular breakfast consumption has been associated with improved weight control, better cognitive function and cardio-metabolic health [1,2,3,4,5]. Recommendations aimed at improving breakfast quality tend to be mainly food based with only a limited number being nutrient based. Food-based recommendations issued by some governments and national dietetic associations list foods that are viewed as integral components of a healthy breakfast which are generally in line with and derived from food-based recommendations for the overall daily diet [6,7,8]. Nutrient-based recommendations typically set breakfast nutrient standards at 20–25% of daily intake recommendations [7,9,10,11,12], given that breakfast generally tends to contribute 20–25% of daily energy intakes. As an example, seeking to update the US National School Breakfast Program, the US Department of Agriculture (USDA) requested the US National Academies of Science, Engineering and Medicine (NASEM) to propose nutrient recommendations for a healthy breakfast in schools. NASEM proposed to apply a figure of 21.5% to the Target Median Intake (TMI) for micronutrients [13]. The figure of 21.5% represented the mid-point of the range for the percent energy from breakfast (19–24%) as measured in the School Nutrition Dietary Assessment Study-III [10,14].

The derivation of breakfast recommendations on nutrient intake and their translation into culturally tailored food based dietary advice can help policy makers, educators and industry to develop better public health nutrition strategies to optimize food choices at breakfast. For example, it could aid these stakeholders with respect to product reformulation, food fortification and in the development of nutrition-based public health policy, such as in public procurement for government funded school breakfasts.

The International Breakfast Research Initiative (IBRI) set out to develop a new approach to define quantitative breakfast nutrient recommendations taking both observed breakfast and daily nutrient intakes into account; similar to NASEM but with a unique focus on breakfast nutrient intakes that were associated with highest overall daily diet quality [15]. Furthermore, the approach utilized data from six countries, with the aim of developing nutrient recommendations for breakfast for Western Europe and North America, that would allow for local adaptation. Dietary intake data from Canada, Denmark, France, Spain, UK, and the US were analyzed following a harmonized approach. The results from these country-specific analyses are presented in this special issue of *Nutrients* [16,17,18,19,20,21].

The present study summarizes the results of these analyses which underpins a proposal of guiding principles for the development of nutrient recommendations for breakfast. The application of these principles led to the development of two sets of nutrient recommendations for breakfast: one for children and adolescents and one for adults.

## 2. Methods

### 2.1. Data Sources

Table 1 outlines the key attributes of dietary databases for the 6 countries. The focus was Western Europe and North America. Final country selection was based on the availability of recent dietary intake data including both children and adults and geographical coverage of Europe. Data were analyzed for children, adolescents, younger adults and older adults. For each, intakes of macronutrients and micronutrients were determined for both breakfast and for the total day. Nutritional supplements were excluded, because of the considerable variation in their use across the six countries and because of the extent to which they skewed micronutrient intakes. Moreover, and in line with dietary guidelines, it was the intention to develop nutrient recommendations for breakfast with an expectation that these standards would be met through foods rather than supplements.

### 2.2. Assessment of Diet Quality

The Nutrient Rich Foods Index (NRF) [28] was adapted to serve as a measure of overall diet quality. Children/adolescents and younger/older adults were separately stratified by NRF tertiles. The NRF was calculated as follows, with nutrient intakes adjusted for 2000 kcal and expressed in percentage of a daily reference value:

NRF=(∑i=19IntakeiEnergy×2000DVi−∑j=13IntakeiEnergy×2000MRVj−1)×100

where Intake_i or j_ is the daily intake of each of the nine nutrients to encourage *i* (protein, fiber, vitamin A, vitamin C, vitamin D, calcium, iron, magnesium, and potassium) or three nutrients to limit *j* (saturated fat, total or added sugar, and sodium), *DV_i_* and *MRV_j_* are the reference daily value and maximum reference value, respectively for nutrients *i* and *j*, and Energy represents daily energy intake. Following past protocol, percent DVs for nutrients to encourage were truncated at 100, so that high intake of one nutrient could not compensate for the dietary inadequacy of another.

Relevant nutritional labelling standards were used as nutrients’ reference values [29,30,31,32]. The advantage of using nutrient values for food labeling is that they are applicable to all ages except toddlers. In contrast, were dietary reference values to be used, this would require the calculation of different indices for different age groups and between males and females, given the fact that these drive variabilities in nutrient requirements. The nutrient intakes at breakfast for those at the upper tertile, i.e., with highest overall diet quality, were used for the development of quantitative guidelines.

### 2.3. Data Compilation

To derive nutrient recommendation applicable to the six countries, a summary of the contribution of breakfast in each country was compiled. There was no further adjustment as the objective was not to statistically compare the contribution of breakfast to nutritional intakes across countries, but to provide indicative ranges. As a result, no analytical statistics on pooled data were performed for this specific study, but results from the statistical analyses of the individual country studies were used to inform the development of the nutrient recommendations. Due to differences in the definition of age groups across countries, it was decided to divide the population into two groups for the recommendations: children and adolescents, and adults.

## 3. Results

### 3.1. Consumption of Breakfast and Its Nutritional Pattern in the 6 Countries

**Figure 1:**
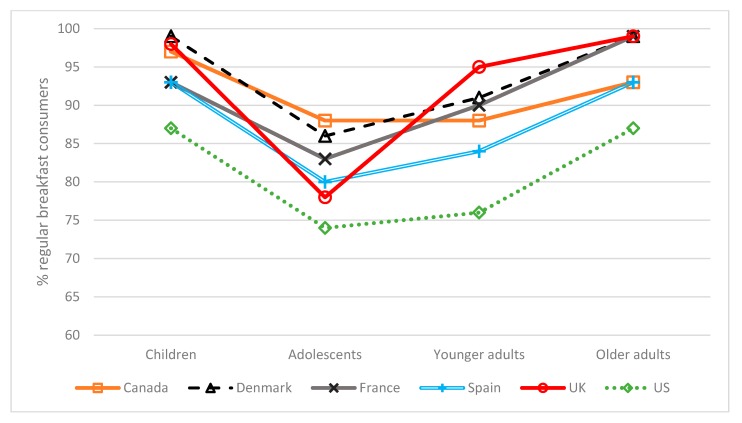
Proportion of regular breakfast consumers by country and age group Regular breakfast consumption was defined as follow: Canada–consumed breakfast on the recall day; Denmark and France–consumed breakfast ≥5 days out of 7; Spain–consumed breakfast 3 out of 3 days; UK-consumed breakfast ≥3 days out of 4; US-consumed breakfast with more than 50 kcal on the recall day. Age groups were defined as follow: Children (6–12 years, except Spain 9–12 years and UK 5–12 years); Adolescents (13–17 years, except UK 13–18 years); Younger adults (18–54 years, except UK 19–64 years); Older adults (55+ years, except UK 65+ years).

The data used varied in the nature of the survey tool used, the period of observation and the exact age ranges in the four different age categories (Table 1). Nonetheless, there was a general pattern for the proportion of the population deemed to be regular breakfast consumers across all six countries. This figure was very high in young children but declined with adolescence, rising thereafter in younger adults and further still in older adults where the values were equivalent or higher to those for children (Figure 1). Overall, the vast majority of the survey populations did report regular breakfast consumption.

**Figure 2:**
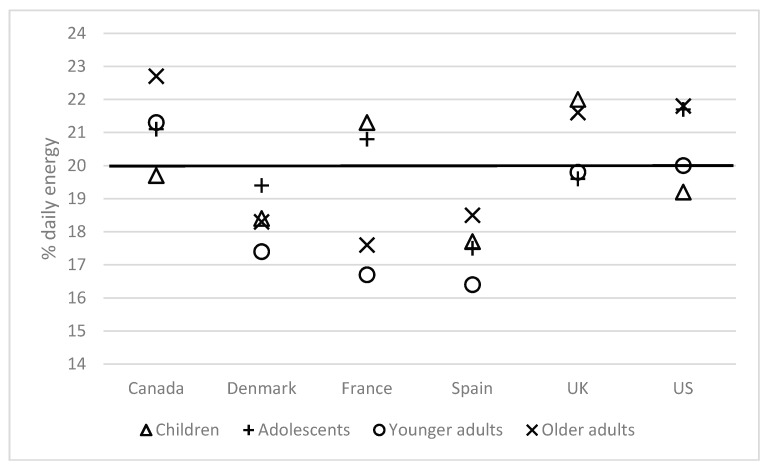
Contribution of breakfast to daily energy among regular breakfast consumers, by country and age group Age groups were defined as follows: Children (6–12 years, except Spain 9–12 years and UK 5–12 years); Adolescents (13–17 years, except UK 13–18 years); Younger adults (18–54 years, except UK 18–64 years); Older adults (55+ years, except UK 65+ years).

**Figure 3:**
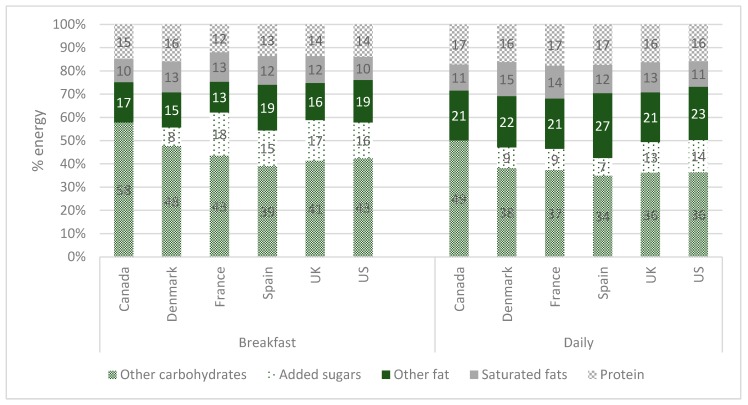
Macronutrient profiles (% energy contributions) of breakfast and daily intakes among regular breakfast consumers, in the total survey populations. Added sugars intake data were not available for Canada; thus “other carbs” are total carbohydrates in the Canada bar.

A key figure used in the literature to study breakfast patterns is the contribution of breakfast to overall energy intake. The figure of 20% is often proposed [7,11,13]. The data in Figure 2 would broadly concur with those previous findings but also illustrate that this figure varies among countries and independently, across age groups. The distribution of macronutrient intakes is shown in Figure 3. For this and subsequent tables, the data are presented for the total survey populations (only including regular breakfast consumers); results per age group, which follow along similar lines, are contained in the Supplementary Material (Figures S1 and S2). 

Notwithstanding the variation across countries in macronutrient intakes, breakfast was a carbohydrate-rich eating occasion relative to the entire day (Figure 3). As a result, the energy contribution of protein and fats were lower at breakfast than for the entire day.

**Figure 4:**
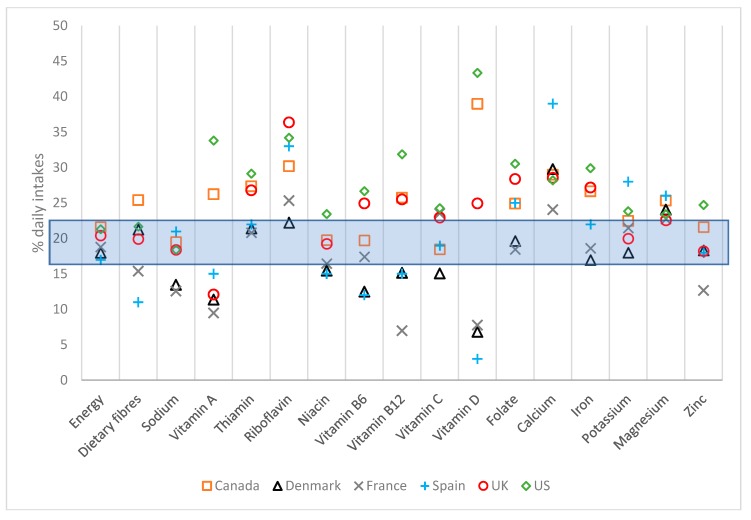
Contribution of breakfast to daily energy and nutrient intakes, total survey populations. The box represents observed contribution of breakfast to daily energy intakes.

The contribution of breakfast to daily energy and micronutrient intakes is compared in Figure 4. Whilst breakfast contribution to daily energy tended to have a low level of variation among the six countries, contributions of breakfast to daily micronutrient intakes showed a much greater degree of variability. However, for several of these micronutrients (thiamine, riboflavin, folate, calcium, potassium and magnesium), all countries showed breakfast contribution to daily intakes that exceeded the relative energy contribution of breakfast. 

### 3.2. Nutritional Intakes at Breakfast among Individuals with Highest Overall Diet Quality

Within each dietary survey, participants were divided into tertiles of daily diet quality, as measured with the NRF. No association was observed between energy intake at breakfast and NRF tertiles in most countries. However, for most micronutrients, except sodium, and for protein and fiber, intakes at breakfast tended to be higher in the upper tertile (Supplementary Table S1, [16,17,18,19,20,21]). In contrast, breakfast intakes of total and saturated fats, sodium and added sugars were lower in the upper tertile in most countries. These results confirmed the important contribution of breakfast to overall daily nutritional quality and indicated that intakes at breakfast of individuals in the upper tertile of NRF could effectively be used as a guide for setting attainable quantitative recommendations that could improve total daily diet quality. Table 2 (children and adolescents) and Table 3 (adults) show nutritional intakes at breakfast among individuals in the upper tertile of the NRF, together with the contribution of these intakes to the appropriate reference food labeling standard used as reference for daily intakes. 

### 3.3. Principles for the Development of Nutrient Recommendation for Breakfast

The following key guiding principles were established to develop nutrient recommendations for breakfast (Table 4). Nutrient intakes for breakfasts associated with the highest-quality diets were related to recommended daily intake from the 2018 Codex international food standards [33]. WHO guidelines were used for nutrients of public health concern (added sugars, saturated fats, salt), and for total carbohydrates [34,35].

**Principle** **1.** *The contribution of breakfast ranged from 16 to 23% of daily energy intake (Figure 1), depending on country and age group. As a result, a value of 20% of the daily recommended value is proposed as a benchmark for setting nutrient recommendations. The default value can be adjusted in the light of any concerns about individual nutrients as outlined in Principles 2, 3, 4 and 5.*

The observed range of energy intakes at breakfast (293–471 kcal) is proposed to set breakfast energy recommendations. While individual dietary intakes at breakfast can vary widely depending on country, age, gender, physical activity level and other factors, the present default value of 300–500 kcal is proposed for healthy, free-living subjects. This converts to 15–25% of daily energy based on a 2000 kcal diet.

**Principle** **2.** *For nutrients where there is evidence that the mean daily population intake is close to the recommended intake among most countries compared to the Codex reference value (Supplementary Table S2), it would appear prudent to set the target closer to the lower range of the mean national values found in the upper NRF tertile. Principle 2 was applied to vitamin A, riboflavin and niacin in both age groups, and to thiamin and vitamin B12 in children and adolescents.*

**Principle** **3.** *For nutrients where (1) the mean population intakes are close to optimal relative to the Codex reference values, and (2) breakfast contributes significantly to daily intakes (>20%), the target is set to the average intakes range from individuals in the upper NRF tertile. Principle 3 was applied to calcium. Principle 3 was also applied to total carbohydrates, as breakfast was a carbohydrate-rich meal in all 6 countries. As a result the WHO recommendation of 55–75% of energy contribution from carbohydrates [35] was used.*

**Principle** **4.** *In contrast, where the mean population intakes are generally less than optimal relative to the Codex reference values and there is wide variation in breakfast contribution across countries, the target was based on 20% of the Codex value, given that across countries, breakfast provides on average, 20% of daily energy needs. Principle 4 was applied to fiber, vitamin D, vitamin C, folate, iron, potassium, magnesium and zinc. For sodium, for which intake is above recommendations in all analysed countries, the same principle applies as a maximum threshold rather than minimum requirement.*

**Principle** **5.** *For macronutrients to limit, the proposal is to adopt the WHO guideline values expressed as % of breakfast energy. This applies to added/free sugars and total and saturated fats, for which the proposed guidelines results in 10% of breakfast energy for sugars and saturated fat, and up to 30% for total fats.*

## 4. Discussion

The IBRI project aimed to derive nutritional recommendations for the breakfast eating occasion based on observed nutritional profiles of breakfasts in six countries (Canada, Denmark, France, Spain, UK and US), taking into account current international dietary guidelines. The analyses of individual countries’ breakfast patterns showed a remarkable level of consistency in the regularity of breakfast consumption, its contribution to daily energy, and macronutrient profiles, with breakfast being consistently a carbohydrate-rich eating occasion. Breakfast’s contribution to daily micronutrient intakes showed significant variability among countries. Nonetheless, breakfast was consistently shown to be a nutrient-rich eating occasion relative to its contribution to daily energy, and a strong contributor to thiamin, riboflavin, folate, calcium, potassium, and magnesium intakes across all countries. 

Based on these observations, the IBRI proposes a set of recommendations for a balanced breakfast. First, the average energy contribution of breakfast is used as reference to set guidelines for other nutrients. Second, specific levels for individual nutrients are adapted based on the nutritional profiles of breakfast among individuals with highest overall daily diet quality and current public health sensitivity of the nutrients. The outcome of the IBRI project is two sets of nutrient recommendations for breakfast: one for children and adolescents, and one for adults and the elderly. Nutrient recommendations are expressed as a percentage of international daily reference values of Codex and WHO. These recommendations aim to ensure that breakfast maintains its current nutrient density while being improved for nutrients of public health concern in Western Europe and North America, e.g., added sugars, saturated fats, fiber and vitamin D. 

A starting point for the IBRI approach was the definition of an optimal daily nutrient intake. To that end, the NRF index was used to provide such a score for each subject’s total dietary intake in each database. This index was chosen because of its emphasis on micronutrients as positive drivers of the scoring system, given the observed significance of breakfast in contributing to micronutrient intake and given that quantitative food group information (on which other diet quality indices such as the Healthy Eating Index are based) was not available in all countries. 

A second issue was to choose an appropriate reference intake to use for expressing the breakfast guidelines. Normally, national Dietary Reference Intakes (DRIs) are used. However, given the international nature of the project and the fact that national DRIs are not always identical across countries nor are they expressed in comparable age ranges, it was decided for consistency to use reference values for nutritional labelling issued by Codex [33] and the diet guidelines of WHO for nutrients without labelling standards [34]. We fully acknowledge that using such international nutrient targets may not be relevant in some countries or some specific populations. The proposed IBRI recommendations are expressed as a percentage of the daily recommendations. As a result, it is possible to tailor these IBRI recommendations by using the most relevant daily national recommendation values to derive the absolute nutrient targets.

The novelty of the IBRI approach lies in the consideration of the specific nutritional profiles of existing breakfast patterns. As detailed in this manuscript, the proposed IBRI nutrient recommendations are not all aligned on the relative contribution of breakfast to energy intakes, i.e., 20% on average, unlike other approaches that derive quantitative recommendations for the breakfast eating occasion [11,13,36]. It is clear from the present study that across countries, intakes of most micronutrients at breakfast in the upper NRF tertile generally exceeded the normal 20% of reference intake (Table 2 and Table 3). Rather than use these values as they stood, it was also necessary to take into account the overall daily population intakes of nutrients, not just breakfast intakes. Thus, for micronutrients, the guiding principles were established (Principles 2–4). 

In the case of a recommendation on energy intake, a single value is not feasible. Individuals may have a higher than average energy intake due to a high level of physical activity and others may need to curb energy intake to control bodyweight. Thus, the mean observed energy intakes at breakfast across age group and countries (293–471 kcal) was adjusted to a range of 15–25% of daily energy, i.e., equivalent to 300–500 kcal for a 2000-kcal diet, similar to the criteria of O’Neil and colleagues [11]. These proposed recommendations could also apply to individuals not consuming breakfast, providing that they shift some of their intake to breakfast, and do not add an extra eating occasion on top of their usual daily intakes as this would likely result in an excess of daily energy intake. 

For macronutrients whose recommendation depends on energy, it is proposed to derive absolute targets based on the observed contribution of breakfast to daily energy or the energy content of breakfast, depending on availability. If such information were not available, it is suggested to consider that breakfast contributes to 20% of the daily recommended energy intake.

The variability in micronutrient intakes observed among the six countries could be partly explained by differences in breakfast patterns and different fortification practices. As an example, milk is commonly fortified with vitamin D in the US [37], and it is a legal requirement in Canada [38]. Similarly, flour is enriched with folic acid, other B vitamins and iron in both countries, and the US permits voluntary fortification in many food categories [39,40]. In comparison, food fortification is much less common in Europe, with mandatory fortification limited to a few categories and countries (e.g., salt iodization is voluntary in most European countries and compulsory in some [41]). As observed with the results from the US and Canada, the fortification of food items commonly consumed at breakfast could be a possible strategy to increase the intakes of specific nutrients at a population level of some key shortfall nutrients, e.g., vitamin D.

The derivation of nutrient recommendations for breakfast will help policy makers and educators develop better public health nutrition strategies to optimize food choices at breakfast. Nutrient targets could be used to guide public procurement of government funded institutions such as schools or hospitals, and for health professionals to better tailor their advice. For the food industry, the proposed recommendations could aid in respect of product reformulation, food fortification or consumer communication.

The results of the present study have some limitations linked to the use of observed dietary patterns from six countries to derive the nutrient recommendations. First, dietary assessment tools differed across countries, as did the study samples. Since the objective was not to compare estimates across countries but rather to have an overall understanding of the nutritional contribution of breakfast, values published in the country-specific studies were used [16,17,18,19,20,21]. Further studies may wish to explore more advanced technique of data compilation, providing availability of data.

In addition, the definition of breakfast differed across countries. Whereas most studies made it clear to participants what to report for breakfast or allowed participants to self-report the identity of an eating occasion (Canada, Denmark, France, Spain, and the US), the UK survey only included time estimates from which breakfast had to be defined. International agreement of a definition of breakfast would considerably assist future research efforts to understand the contribution of this meal occasion to overall dietary intakes.

Furthermore, the proposed age grouping for the nutrient recommendations may not reflect evolving nutrient requirement during childhood. The structure of the different samples varied across countries, with different definitions of age groups. This did not allow recommendations for more detailed age groups to be derived as these differences may have influenced the proposed recommendations. The choice of a single age threshold appeared more consistent with the analyzed surveys in which the threshold to define the adult population was 18 years in all countries but the UK (19 years).

The main strength of the IBRI lies in the consideration of the nutritional profiles of breakfast from six countries. This allowed the IBRI to propose nutrient recommendations adapted to the current patterns of breakfast in the countries studied. While the nutrient recommendations proposed in this study should not be applied to countries and regions where breakfast patterns may be very different, the approach developed by IBRI would be replicable in other regional settings with distinct dietary patterns and where the nutritional profiles of breakfast may be very different, e.g., in Asian or African populations. In effect, while the values proposed in the present study are of value to stakeholders, the methodology used to derive these values is an equally important outcome of the present study.

The variations in food intakes at breakfast outlined in the individual country studies [16,17,18,19,20,21] highlight the need for food-based breakfast guidelines to be made on a country level. In deriving such food-based guidelines, specific emphasis would be required for nutrients with clear gaps between current intakes and recommended values—at breakfast or daily, e.g., for fiber, vitamin D and added sugars.

## 5. Conclusions

The International Breakfast Research Initiative set out to develop a methodology for the establishment of quantitative nutrient recommendations for breakfast and to do so in the context of six countries (Canada, Denmark, France, Spain, UK, and the US). The novelty of the IBRI approach lies in the combined consideration of the specific nutritional profiles of existing breakfast patterns and daily requirements. The proposed nutrient recommendations presented in this study, while being applicable only in countries with predominantly Western diet patterns, can be tailored to specific target population by using the relevant daily recommended intake values. In addition, the methodology developed can be adopted on a country by country basis providing that national dietary intake data are available. Developing breakfast-specific nutrient recommendations can guide the development of more meaningful public health nutrition strategies to optimize food choices at breakfast and can ultimately help consumers make healthier choices at breakfast.

## Figures and Tables

**Table 1:** Dietary intake surveys used in the International Breakfast Research Initiative (IBRI), and associated definition of breakfast in the surveys.

Country	Study Name, Year and Reference	Diet Assessment Method	Breakfast Definition	Number of Participants with Complete Dietary Information			
				Children (6–12 Years)	Adolescents (13–17 Years)	Younger Adults (18–54 Years)	Older Adults (55+ Years)
Canada	CCHS-Nutrition 2015 [22]	1-day 24 h recall	Self-defined	2331	2026	7631	6279
Denmark	Danish national survey of diet and physical activity 2011-2013 [23]	7-day food record	Section in questionnaire	476	272	1791	1118
France	CCAF 2013 [24]	7-day food record	Self-defined	426	250	595	445
Spain ^$^	ANIBES 2013 [25]	3-day diet recall	Section in questionnaire	213	211	1655	206
UK *	NDNS 2008-14 [26]	4-day estimated food diary	All items consumed between 6–11 a.m.	1947	1534	3619	1074
US	NHANES 2011-14 [27]	1-day 24 h recall	Self-defined. Intake > 50 kcal	2511	1546	6594	3837

* Age groups in UK: 5–12 years, 13–18 years, 19–64 years, 65+ years. ^$^ Age groups in Spain: 9–12 years for children. CCAF: Comportements et consommations alimentaires en France; NDNS: National Diet and Nutrition Survey; ANIBES: Antropometría, Ingesta y Balance Energético en España; NHANES: National Health and Nutrition Survey; CCHS: Canadian Community Health Survey.

**Table 2:** Nutritional intakes at breakfast, among children and adolescents aged 6–17 years in the third (upper) tertile of the Nutrient Rich Foods (NRF) diet quality index.

Nutrient	Breakfast Intakes in NRF T3 (Quantity)						Intakes as % Breakfast Energy or % NRV ^1^					
	Canada	Denmark	France	Spain	UK ^4^	US	Canada	Denmark	France	Spain	UK ^4^	US
Energy (kcal)	368	387	384	348	347	366						
Total Carbohydrates (g) ^1^	56	62	60	47	54	57	61.5	62.5	63.0	55.0	61.0	65.0
Added sugars (g) ^1,2^	n/a	6.6	16.4	12.4	13	12.2	n/a	6.8	16.0	15.5	14.0	14.0
Total Fat (g) ^1^	10.4	9.2	10.8	11.6	10	10.1	23.1	21.3	25.0	31.0	25.0	23.0
Saturated Fat (g) ^1^	4	3.9	5.4	5	4	4	9.4	9.1	12.5	15.6	11.0	10.0
Protein (g) ^1^	14.4	15.7	11.8	11.5	12	13.7	28.8	31.3	23.4	23.0	24.0	27.4
Fiber ^3^ (g)	4.1	5	2.8	1.3	3.7	3.4	16.2	20.0	11.2	5.4	14.8	13.6
Sodium (mg)	424	386	350	385	356	454	21.2	19.3	16.9	19.3	17.8	22.7
Vitamin A (mg)	181	102	80	57	107	294	22.6	12.8	10.1	7.1	13.4	36.8
Thiamin (mg)	0.5	0.3	0.4	0.3	0.4	0.6	43.3	26.7	33.3	20.8	33.3	50.0
Riboflavin (mg)	0.6	0.5	0.6	0.5	0.7	0.9	52.5	40.8	50.0	41.7	58.3	75.0
Niacin (mg)	6.4	4.0	3.8	4.1	7	6.3	42.4	26.5	25.3	27.3	46.7	42.0
Vitamin B6 (mg)	0.4	0.3	0.5	0.3	0.6	0.7	27.7	19.2	38.5	23.1	46.2	53.8
Vitamin B12 (mcg)	1.2	1.1	0.6	0.7	1.4	2.4	50.8	45.0	25.0	29.2	58.3	100.0
Vitamin C (mg)	20.7	16.0	28	8.5	22	28	20.7	16.0	33.0	8.5	22.0	28.0
Vitamin D (mcg)	2.5	0.3	0.2	0.2	0.7	3.4	50.8	5.0	4.0	4.8	14.0	68.0
Folate (mcg)	95	64	67	38	79	167	23.9	16.0	17.5	9.5	19.8	41.8
Calcium (mg)	328	339	320	304	260	370	32.8	33.9	32.1	30.4	26.0	37.0
Iron (mg)	4.1	2.2	3.5	2	3.3	6.3	29.0	15.6	24.3	14.3	23.6	45.0
Potassium (mg)	590	600	630	541	523	581	16.9	17.1	18.3	15.5	14.9	16.6
Magnesium (mg)	72	99	73	42	53	63	23.4	31.9	23.5	13.5	17.1	20.3
Zinc (mg)	2.2	2.2	1.6	1.2	1.5	3.5	20.4	19.8	14.5	10.9	13.6	31.8

NRV: Nutrient Reference Value. ^1^ In the second set of columns, macronutrients (total carbohydrates, added sugars, total fat, saturated fat) intakes are expressed as percent energy; for other nutrients, intakes are expressed as percent NRV (see NRV values in Table 4). ^2^ Added sugars’ definitions vary by country: non-milk extrinsic sugars measured in UK; free sugar values in France; no data available in Canada. ^3^ Fiber intakes in the UK are measured as non-starch polysaccharides and multiplied by 1.33 to convert to fiber values. ^4^ Age ranges: UK (5–18 years).

**Table 3:** Nutritional intakes at breakfast, among adults aged 18+ years in the third (upper) tertile of the NRF diet quality index.

Nutrient	Breakfast Intakes in NRF T3 (Quantity)						Intakes as % Breakfast Energy or % NRV ^1^					
	Canada	Denmark	France	Spain	UK ^4^	US	Canada	Denmark	France	Spain	UK ^4^	US
Energy (kcal)	381	402	343	290	325	415						
Total Carbohydrates (g) ^1^	57.4	61	54.6	39.2	52	63.3	57.4	58.4	63.6	56.2	61.0	62.9
Added sugars (g) ^1,2^	n/a	6.8	12.4	6.2	11	11.6	n/a	6.5	16.0	11.6	12.0	11.5
Total Fat (g) ^1^	11.4	11.2	9.5	8.8	9	12.3	24.4	24.5	24.2	28.4	24.0	24.6
Saturated Fat (g) ^1^	3.7	4.7	4.5	3.1	3	4	8.2	10.3	11.5	11.7	9.0	8.2
Protein (g) ^1^	15	16.8	9.9	10.1	12	16.4	30.0	33.6	19.8	20.2	24.0	32.8
Fiber ^3^ (g)	6	5.8	3.3	1.8	4.6	5.5	23.8	23.2	13.2	7.0	18.4	22.2
Sodium (mg)	423	409	400	293	337	507	21.2	20.5	20.0	14.7	16.9	25.4
Vitamin A (mg)	166	112	81.6	27.7	97	306	20.8	14.0	10.2	3.5	12.1	38.3
Thiamin (mg)	0.5	0.3	0.2	0.2	0.4	0.6	40.8	24.2	16.7	17.5	33.3	46.7
Riboflavin (mg)	0.6	0.5	0.4	0.4	0.6	0.9	49.2	37.5	33.3	33.3	50.0	75.8
Niacin (mg)	7.3	5.6	3.3	3.7	7	6.6	48.5	37.2	22.0	24.7	46.7	43.8
Vitamin B6 (mg)	0.4	0.3	0.3	0.2	0.5	0.8	29.2	19.2	23.1	16.2	38.5	62.3
Vitamin B12 (mcg)	1.0	1.0	0.4	0.5	1.3	2.3	39.6	42.1	16.7	21.3	54.2	95.8
Vitamin C (mg)	28	21	22	10	23	35	27.8	21.4	21.8	10.0	23.0	34.7
Vitamin D (mcg)	1.8	0.4	0.2	0.1	0.8	2.8	36.0	8.2	4.0	1.0	16.0	55.8
Folate (mcg)	103	72.2	49.3	29	76	265	25.8	18.1	12.3	7.4	19.0	66.3
Calcium (mg)	262	357	228	221	232	348	26.2	35.7	22.8	22.1	23.2	34.8
Iron (mg)	3.8	2.2	2.5	1.7	3	6.8	26.9	15.6	17.9	11.9	21.4	48.5
Potassium (mg)	695	726	665	477	637	789	19.9	20.7	19.0	13.6	18.2	22.5
Magnesium (mg)	96.1	111	82.3	46.6	68	95.3	31.0	35.8	26.5	15.0	21.9	30.7
Zinc (mg)	2.4	2.4	1.5	1.2	1.6	3.7	21.3	21.7	13.6	10.6	14.5	33.9

NRV: Nutrient Reference Value. ^1^ In the second set of columns, macronutrients (total carbohydrates, added sugars, total fat, saturated fat) intakes are expressed as percent energy; for other nutrients, intakes are expressed as percent NRV (see NRV values in Table 4). ^2^ Added sugars definitions vary by country: non-milk extrinsic sugars measured in UK; free sugar values in France; no data available in Canada. ^3^ Fiber intakes in the UK are measured as non-starch polysaccharides and multiplied by 1.33 to convert to fiber values. ^4^ Age ranges: UK 19+ years.

**Table 4:** Proposed nutrient recommendations for the breakfast eating occasion.

Nutrient	Children and Adolescents	Adults	World Health Organization (WHO)/CODEX International Food Standards Daily Recommended Value [33,34,35]
**Principle 1**			
Energy (kcal)	300–500	300–500	
**Principle 2**			
Protein (% NRV)	>20	>20	50 g
Vitamin A (% NRV)	>10	>10	800 mg
Thiamin (B1) (% NRV)	>25	>20	1.2 mg
Riboflavin (B2) (% NRV)	>35	>30	1.2 mg
Niacin (B3) (% NRV)	>25	>25	15 mg
Vitamin B6 (% NRV)	>20	>20	1.3 mg
Vitamin B12 (% NRV)	>25	>20	2.4 mcg
**Principle 3**			
Calcium (% NRV)	>30	>25	1000 mg
Total carbohydrates (%en)	55–75	55–75	55–75 %energy
**Principle 4**			
Fiber (% NRV)	>20	>20	25 g
Vitamin C (% NRV)	>20	>20	100 mg
Vitamin D (% NRV)	>20	>20	5 mcg
Folate (% NRV)	>20	>20	400 mcg
Iron (% NRV)	>20	>20	14 mg
Potassium (% NRV)	>20	>20	3500 mg
Magnesium (% NRV)	>20	>20	310 mg
Zinc (% NRV)	>20	>20	11 mg
Sodium (% NRV)	<20	<20	2000 mg
**Principle 5**			
Added sugars (%en)	<10	<10	<10 %energy
Total Fat (%en)	20–30	20–30	<30 %energy
Saturated Fat (%en)	<10	<10	<10 %energy

NRV: Nutrient Reference Value; %en: % breakfast energy.

## Supplementary Materials

The following are available online at http://www.mdpi.com/2072-6643/10/10/1540/s1, Figure S1. Macronutrient profiles of breakfast and daily intakes, by age group; Figure S2. Contribution of breakfast to daily intakes, by age group; Figure S3. Governing Principles document signed by all authors not employees of CPW SA or General Mills Inc.; Table S1. Trends for nutritional intakes at breakfast across tertiles of NRF; Table S2. Average daily nutrient intakes expressed as % energy (%en) or % daily recommended value (DRV).
