# Supplementary material for: Towards an Evidence-Based Recommendation for a Balanced Breakfast—A Proposal from the International Breakfast Research Initiative
Source: Nutrients. 2018 Oct 18;10(10):1540. doi: 10.3390/nu10101540 (PMC6213610; doi:10.3390/nu10101540)
Supplement: Supplementary file 1 [file nutrients-10-01540-s001.zip › nutrients-357826-SI.pdf]

Towards an evidence-based recommendation for a balanced breakfast - a proposal from the International Breakfast Research Initiative

Supplementary materials

Figure S1. Macronutrient profiles of breakfast and daily intakes, by age group

Figure S2. Contribution of breakfast to daily intakes, by age group

Table S1. Trends for nutritional intakes at breakfast across tertiles of NRF

Table S2. Average daily nutrient intakes expressed as %energy (%en) or % daily recommended value (DRV)

Figure S3. Governing Principles document signed by all authors not employees of CPW SA or General Mills Inc.

Figure S1 – Macronutrient profiles of breakfast and daily intakes.

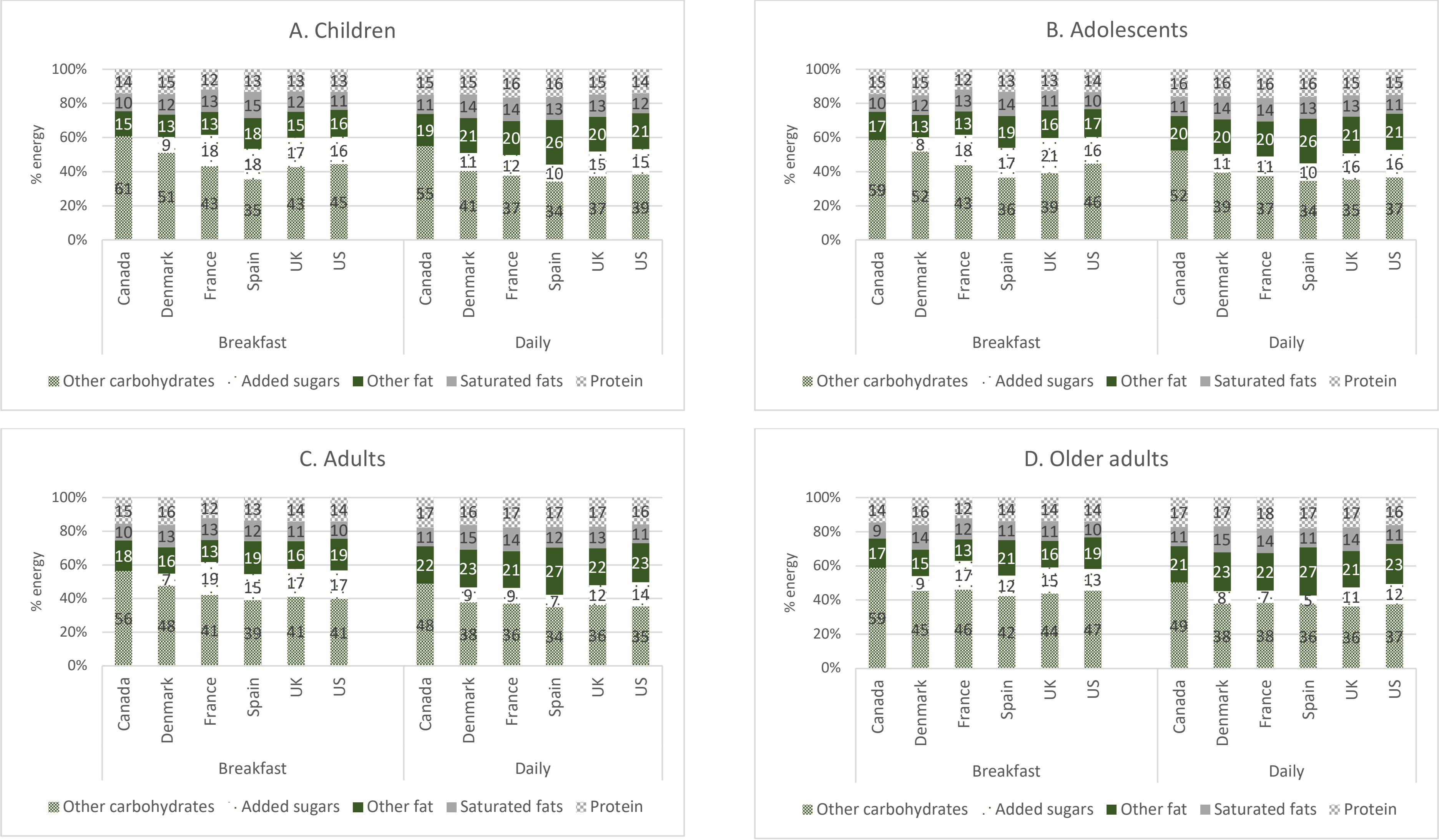

Added sugar data was not available in the Canadian food composition table; thus “Other carbohydrates” represent total carbohydrates.

Figure S2 - % contribution of breakfast to daily intakes

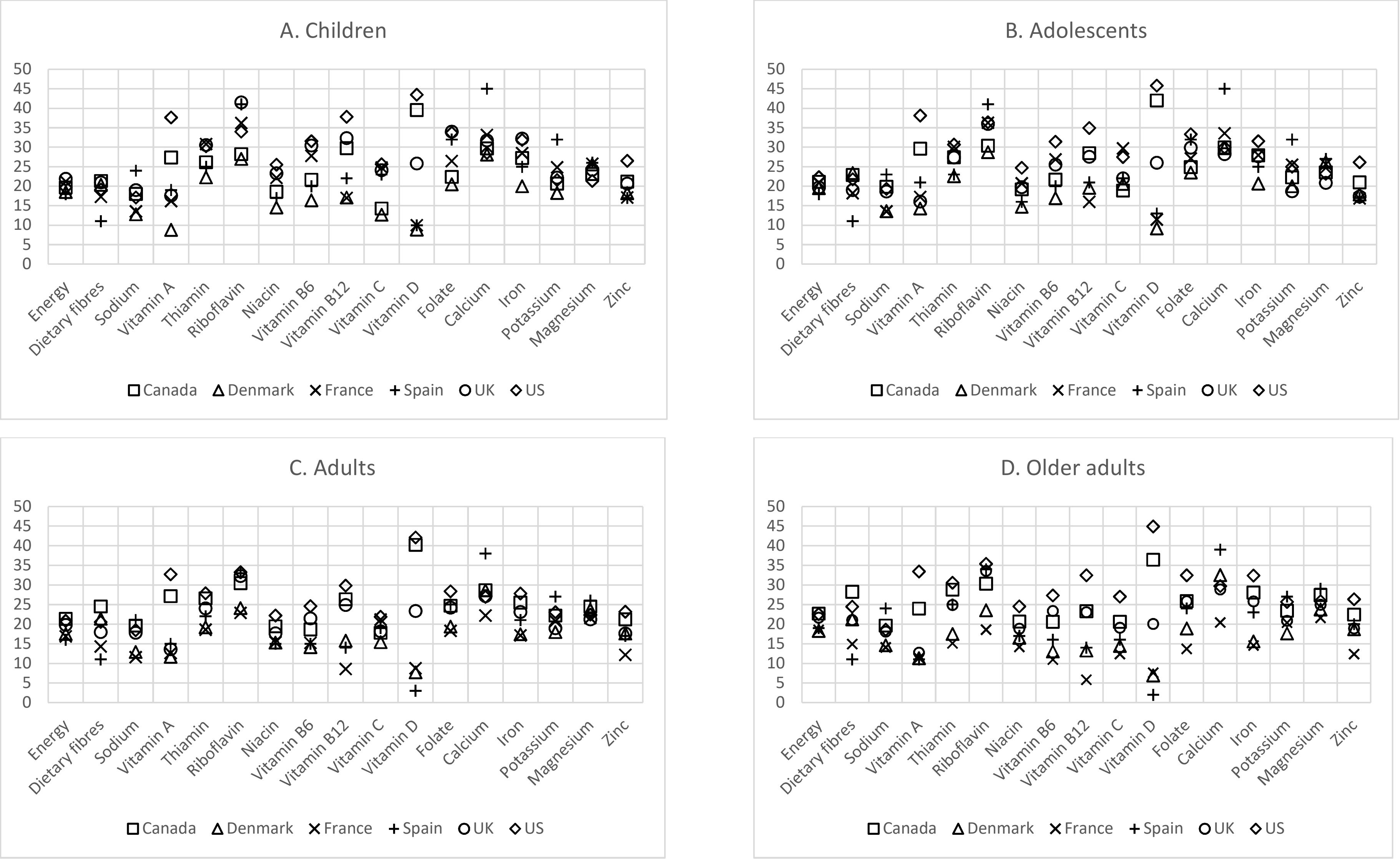

Table S1. Trends for nutritional intakes at breakfast across tertiles of NRF

	Children and adolescents (6-17 years)						Adults (18+ years)					
**Nutrient**	**Canada**	**Denmark**	**France**	**Spain**	**UK[1]**	**US**	**Canada**	**Denmark**	**France**	**Spain**	**UK[1]**	**US**
Energy	-	D***	-	-	-	D***	-	D***	-	-	D*	D***
Total carbohydrates	U**	U***	-	U*	U***	U***	U***	U***	-	U***	U***	U***
Added sugars	n/a	D***	D***	D***	D***	D***	n/a	D***	D***	D***	D***	D***
Total Fat	-	D***	-	D***	D***	D***	D***	D***	D**	D***	D***	D***
Sat Fat	-	D***	-	D***	D***	D**	D***	D***	D***	D***	D***	D***
Protein	-	U***	U***	U**	U***	U***	U**	U***	-	U***	U***	U***
Fiber	U***	U***	U***	U***	U***	U***	U***	U***	U***	U***	U***	U***
Sodium	D*	D***	U***	-	D***	D***	D***	D***	-	-	D***	D***
Vitamin A	U**	D***	U***	U**	U***	U***	U***	D***	-	-	-	U***
Thiamin	U***	U***	U***	U***	U***	U***	U***	U***	U***	U***	U***	U***
Riboflavin	U***	U***	U***	U***	U***	U***	U***	U***	U***	U***	U***	U***
Niacin	-	U***	U***	U***	U***	U***	U**	U***	U***	U***	U***	U***
Vitamin B6	U***	U***	U***	U***	U***	U***	U***	U***	U***	U***	U***	U***
Vitamin B12	U***	U***	U***	U***	U***	U***	U***	-	U***	U***	U***	U***
Vitamin C	U*	U***	U**	U***	U***	U***	U***	U***	U***	U***	U***	U***
Vitamin D	U***	-	-	U***	U***	U***	U***	U***	-	-	U***	U***
Folate	-	U***	U***	U***	U***	U***	-	U***	U***	U***	U***	U***
Calcium	U***	U***	U***	U***	U***	U***	U***	U***	U***	U***	U***	U***
Iron	U***	U***	U***	U***	U***	U***	U***	U***	U***	U***	U***	U***
Potassium	U***	U***	U***	U***	U***	U***	U***	U***	U***	U***	U***	U***
Magnesium	U***	U***	U***	U***	U***	U***	U***	U***	U***	U***	U***	U***
Zinc	U***	U***	U***	U**	U***	U***	U***	U***	U***	U***	U***	U***

NRF: Nutrient Rich Food Index [28]; U: Upwards trend (positive association); D: downwards trend (negative association); -: no trend; p-values for fully-adjusted models: *p<0.1, **p<0.05, ***p<0.01

[1] Age ranges in the UK data: 5-18 years and 19+ years.

Table S2. Average daily nutrient intakes expressed as %energy (%en) or % daily recommended value (DRV)[1]

		Canada				Denmark				France			
	DRV[1]	Children	Adolescents	Adults	Older adults	Children	Adolescents	Adults	Elderly	Children	Adolescents	Adults	Older adults
Energy (kcal)		1848	2065	1958	1733	2069	2161	2397	2294	1656	1935	1892	1862
Total Carbohydrates	50-70%en	54.9	52.1	47.4	48.3	51.0	50.4	46.6	45.3	49.2	47.9	45.2	45.4
Added sugars[2]	<10%en					10.5	11.1	8.9	7.5	12.0	11.3	9.1	7.2
Total Fats	<30%en	30.2	31.7	32.5	31.6	34.3	33.9	37.1	38.0	33.5	33.6	35.1	36.2
Saturated Fats	<10%en	10.9	10.9	10.5	10.3	13.8	13.7	14.6	15.3	13.8	13.5	13.8	14.2
Protein	50g	137	164	168	143	152	170	195	192	134	161	166	168
Fibre	25g	63	66	68	69	79	75	90	92	53	61	64	64
Sodium	2000mg	129	146	144	125	161	177	197	182	112	130	146	1
Vitamin A	800mg	74	81	79	81	149	122	124	172	69	77	94	128
Thiamin (B1)	1.2mg	135	149	136	123	108	108	117	117	88	100	92	89
Riboflavin (B2)	1.2mg	151	164	164	150	133	142	150	150	125	140	128	131
Niacin (B3)	15mg	215	266	277	236	159	181	233	235	93	113	120	115
Vitamin B6	1.3mg	107	126	135	119	100	108	131	123	113	130	121	123
Vitamin B12	2.4mcg	156	172	173	159	225	221	275	308	154	181	205	277
Vitamin C	100mg	116	116	101	93	103	94	114	117	78	83	79	99
Vitamin D	5mcg	105	110	92	97	54	58	78	114	34	41	50	51
Folate	400mcg	111	122	117	99	72	71	89	88	54	61	61	69
Calcium	1000mg	93	98	81	73	105	108	116	107	84	93	90	94
Iron	14mg	89	97	90	84	68	70	84	82	72	84	82	86
Potassium	3500mg	68	74	77	76	79	82	31	105	63	71	78	85
Magnesium	310mg	81	94	101	96	97	101	40	123	75	86	99	103
Zinc	11mg	82	98	99	89	92	102	40	110	71	84	87	91

[1] Total carbohydrates, added sugars, total fats and saturated fats expressed as % energy, following WHO guidelines [34,35]; all other nutrients expressed as % DRV, following CODEX Nutrient reference values [33].

[2] Added sugars data was not available for Canada.

Age groups were defined as follow: Children (6-12y, except Spain 9-12y and UK 5-12y); Adolescents (13-17y, except UK 13-18y); Younger adults (18-54y, except UK 19-64y); Older adults (55+y, except UK 65+y)

Table S1 (Continued). Average daily nutrient intakes expressed as %energy (%en) or % daily recommended value (DRV)[1]

		Spain				UK				US			
	**DRV[1]**	**Children**	**Adolescents**	**Adults**	**Elderly**	**Children**	**Adolescents**	**Adults**	**Elderly**	**Children**	**Adolescents**	**Adults**	**Elderly**
Energy (kcal)		1936	1989	1782	1591	1593	1797	1719	1599	1977	2092	2359	1979
Total Carbohydrates	50-70%en	43.6	44.4	40.6	40.6	51.8	50.9	48.0	47.4	53.9	53.5	48.9	48.8
Added sugars	<10%en	9.8	10.1	7.1	5.1	14.6	15.6	11.9	11.1	15.1	16.4	14.1	11.7
Total Fats	<30%en	39.1	38.3	38.8	37.5	33.5	33.9	34.7	35.1	33.0	32.5	33.7	34.0
Saturated Fats	<10%en	13.2	12.6	11.8	10.7	13.1	12.5	12.8	13.8	11.8	11.1	10.8	10.8
Protein	50g	153	157	147	133	115	134	145	136	140	159	182	153
Fibre	25g	46	45	48	56	59	63	72	71	59	61	75	72
Sodium	2000mg	77	79	69	59	95	112	110	100	155	174	193	162
Vitamin A	800mg	34	30	30	28	78	81	113	146	83	78	87	92
Thiamin (B1)	1.2mg	68	71	63	56	110	117	118	117	136	144	150	132
Riboflavin (B2)	1.2mg	94	88	82	78	125	121	129	135	173	178	199	179
Niacin (B3)	15mg	137	146	140	121	183	222	234	209	146	166	194	160
Vitamin B6	1.3mg	73	75	72	69	135	159	162	151	137	152	189	158
Vitamin B12	2.4mcg	124	115	126	123	170	176	210	249	212	224	236	217
Vitamin C	100mg	25	22	28	35	84	78	79	77	80	72	85	88
Vitamin D	5mcg	22	22	28	28	41	44	56	66	122	118	102	100
Folate	400mcg	23	22	21	21	49	53	62	61	138	144	152	137
Calcium	1000mg	63	58	49	47	81	78	80	81	111	112	108	93
iron	14mg	51	51	48	45	63	69	74	70	106	111	118	108
Potassium	3500mg	45	43	42	43	63	67	78	78	65	68	82	79
Magnesium	310mg	47	46	46	45	63	68	81	76	78	84	107	99
Zinc	11mg	55	57	55	49	60	68	76	74	94	102	113	99

[1] Total carbohydrates, added sugars, total fats and saturated fats expressed as % energy, following WHO guidelines [34,35]; all other nutrients expressed as % DRV, following CODEX Nutrient reference values [33].

Age groups were defined as follow: Children (6-12y, except Spain 9-12y and UK 5-12y); Adolescents (13-17y, except UK 13-18y); Younger adults (18-54y, except UK 19-64y); Older adults (55+y, except UK 65+y)

Governing Principles

You, ________________________________ (name), have been engaged by CPW SA based on your topic-expert credentials to participate in research work with the aim to establish a multi-disciplinary expert recommendation on the nutrient and food criteria of a balanced breakfast for European and North American children and adults (the "Project").

The Project is being structured and these governing principles are being issued to ensure the scientific integrity of the Project research work and the resulting publication.

These principles supplement the separate agreement(s) that you have or will enter into to govern your participation in the Project. We kindly ask you to review them carefully and to confirm below that you understand and agree to them.

We, as CPW SA, expect the following governing principles to be respected and adhered to by all participants in the Project for the duration of the Project:

- Participants are expected to provide impartial and objective input throughout the Project.
- Participants are under no obligation to promote or communicate about CPW SA, its products or the breakfast cereal category.
- To the extent a participant makes public statements or other comments (e.g., on social media) regarding CPW SA or its products, he/she shall disclose his/her material connection to the Company.
- All participants will decide on authorship and intellectual input into the scientific paper, but it is anticipated that the paper will be drafted by the Project Chair and Co-Chair with iterative inputs from the other participants. The Project Chair will submit the final paper for publication.
- The resulting publication shall accurately reflect the funding by CPW of the Project. In case a CPW SA employee materially participates in the drafting of the paper, this shall also be accurately reflected.

By signing below, you agree that you understand and agree to be bound by the above governing principles.

Signature: ____________________________

Name: ____________________________

Place and date: ____________________________

Figure S3. Governing Principles document signed by all authors not employees of CPW SA or General Mills Inc.
